# Bioavailability of D-methionine relative to L-methionine for nursery pigs using the slope-ratio assay

**DOI:** 10.7717/peerj.2368

**Published:** 2016-09-07

**Authors:** Changsu Kong, Jong Young Ahn, Beob G. Kim

**Affiliations:** 1 Department of Animal Science and Technology, Konkuk University, Seoul, Republic of Korea; 2 Monogastric Animal Feed Research Institute, Konkuk University, Seoul, Republic of Korea

**Keywords:** Pigs, Methionine isomers, Relative bioavailability, Slope-ratio assay, Nitrogen balance

## Abstract

This experiment was conducted to determine the bioavailability of D-methionine (Met) relative to L-Met for nursery pigs using the slope-ratio assay. A total of 50 crossbred barrows with an initial BW of 13.5 kg (SD = 1.0) were used in an N balance study. A Met-deficient basal diet (BD) was formulated to contain an adequate amount of all amino acids (AA) for 10–20 kg pigs except for Met. The two reference diets were prepared by supplementing the BD with 0.4 or 0.8 g L-Met/kg at the expense of corn starch, and an equivalent concentration of D-Met was added to the BD for the two test diets. The pigs were adapted to the experimental diets for 5 d and then total but separated collection of feces and urine was conducted for 4 d according to the marker-to-marker procedure. Nitrogen intakes were similar across the treatments. Fecal N output was not affected by Met supplementation regardless of source and consequently apparent N digestibility did not change. Conversely, there was a negative linear response (*P* < 0.01) to Met supplementation with both Met isomers in urinary N output, which resulted in increased retained N (g/4 d) and N retention (% of intake). No quadratic response was observed in any of the N balance criteria. The estimated bioavailability of D-Met relative to L-Met from urinary N output (g/4 d) and N retention (% of intake) as dependent variables using supplemental Met intake (g/4 d) as an independent variable were 87.6% and 89.6%, respectively; however, approximately 95% of the fiducial limits for the relative bioavailability estimates included 100%. In conclusion, with an absence of statistical significance, the present study indicated that the mean relative bioequivalence of D- to L-Met was 87.6% based on urinary N output or 89.6% based on N retention.

## Introduction

Crystalline amino acids (AA) are commonly used to provide indispensable AA (also known as essential AA), which limit growth of pigs when protein sources are marginally used to reduce feed cost as well as N excretion. Methionine is essential for protein synthesis and is one of the most limiting AA for the growth of nursery pigs fed diets containing dried blood products (plasma and cells) and dried whey (Cromwell, 2004). Methionine is often supplemented as a racemic mixture of D- and L-Met which is produced through chemical synthesis (Hoehler, Rademacher & Mosenthin, 2005), but only L-Met can be directly used for protein synthesis. Therefore, the conversion of D- to L-Met is indispensible for the utilization of D-Met for protein synthesis in pigs (Dibner & Knight, 1984). Thus, it has been questioned whether the bioefficacy of D- and L-Met for pigs is equal or not. Little research has been conducted to compare the bioefficacy of both Met isomers for pigs and the results have been inconsistent when the growth performances were employed as major measurements (Reifsnyder, Young & Jones, 1984; Chung & Baker, 1992; Shen, Weaver & Kim, 2014). The classical N balance technique measures a N retention rate as an index of protein metabolism (Haymond, 1999) and is more sensitive and more powerful than growth assays. Recently, the bioefficacy of DL-Met relative to L-Met based on N balance was reported (Kong et al., 2016) to the authors’ knowledge, there have been no published reports on the bioavailability of D-Met for pigs using N balance technique. Therefore, it was hypothesized that supplementation of L-Met would have better effects on N balance of pigs compared with D-Met and the present study was conducted to determine the relative bioavailability of D- to L-Met in nursery pigs using the slope-ratio assay.

## Materials and Methods

The Institutional Animal Care and Use Committee of Konkuk University reviewed and approved all protocols (KU13188) used in the present study.

### Animals and experimental design

A total of 50 crossbred ((Landrace × Yorkshire × Duroc) × Duroc) nursery barrows at 35 ± 3 d of age were selected based on BW which was similar across the pigs. For adaptation to metabolism cages, the pigs were individually placed in the cages for five days. After adaptation, the pigs (13.5 ± 1.0 kg) were used to estimate the relative bioavailability of D- to L-Met during five consecutive periods. In each 9-d period, 10 pigs were allotted to five dietary treatments with two replicates per treatment in a randomized complete block design based on the initial BW.

### Diets

A Met-deficient basal diet (BD) was formulated to meet or exceed the estimated requirements of all nutrients except for Met. The BD contained total Met at 18.1 g/kg which was about 66% of the Met requirement for 10–20 kg pigs (NRC, 1998; Table 1). Two reference diets were prepared by supplementing the BD with 0.4 or 0.8 g L-Met/kg at the expense of corn starch, and an equivalent concentration of D-Met was added to the BD for two test diets. To avoid any possible variation in the experimental diets caused by feed ingredients, the experimental diets were made of the same batch of ingredients, mixed at the same time and at the same location. To minimize orts, daily feed allowance was calculated as 3.5% of the BW of each animal at the beginning of each period. The feed was divided into two equal meals and fed to the pigs at 900 and 1,700 h. The pigs were provided ad libitum access to water.

**Table 1:** Ingredient and chemical composition of experimental diets fed to pigs (as-fed basis).

Item^†^	Basal diet	Supplemental L-Met, %		Supplemental D-Met, %	
		0.04	0.08	0.04	0.08
Ingredient composition, %					
Ground corn	55.00	55.00	55.00	55.00	55.00
Dried whey	10.00	10.00	10.00	10.00	10.00
Spray dried animal plasma	10.00	10.00	10.00	10.00	10.00
Corn starch	19.92	19.88	19.84	19.88	19.84
Soybean oil	2.00	2.00	2.00	2.00	2.00
L-Met	–	0.04	0.08	–	–
D-Met	–	–	–	0.04	0.08
L-Lys·HCl	0.32	0.32	0.32	0.32	0.32
L-Thr	0.04	0.04	0.04	0.04	0.04
L-Trp	0.03	0.03	0.03	0.03	0.03
L-Ile	0.14	0.14	0.14	0.14	0.14
Dicalcium phosphate	0.67	0.67	0.67	0.67	0.67
Ground limestone	1.18	1.18	1.18	1.18	1.18
Salt	0.20	0.20	0.20	0.20	0.20
Vitamin-mineral premix^‡^	0.50	0.50	0.50	0.50	0.50
Calculated composition					
Metabolizable energy, kcal/kg	3,552	3,551	3,549	3,551	3,549
CP, %	14.08	14.10	14.13	14.10	14.13
Ether extract, %	4.48	4.48	4.48	4.48	4.48
Met, %	0.18	0.22	0.26	0.22	0.26
Cys, %	0.41	0.41	0.41	0.41	0.41
Choline, %	0.32	0.32	0.32	0.32	0.32
Ca, %	0.72	0.72	0.72	0.72	0.72
Available P, %	0.34	0.34	0.34	0.34	0.34

**Notes:**

† Cys, cysteine; Met, methionine; Lys, lysine; Thr, threonine; Trp, tryptophan; Ile, isoleucine.

‡ Provided the following quantities per kg of complete diet: vitamin A, 25,000 IU; vitamin D_3_, 4,000 IU; vitamin E, 50 IU; vitamin K, 5.0 mg; thiamin, 4.9 mg; riboflavin, 10.0 mg; pyridoxine, 4.9 mg; vitamin B_12_, 0.06 mg; pantothenic acid, 37.5 mg; folic acid, 1.10 mg; niacin, 62 mg; biotin, 0.06 mg; Cu, 25 mg as copper sulfate; Fe, 268 mg as iron sulfate; I, 5.0 mg as potassium iodate; Mn, 125 mg as manganese sulfate; Se, 0.38 mg as sodium selenite; Zn, 313 mg as zinc oxide; butylated hydroxytoluene, 50 mg.

### Sample collection

For the N-balance study, the pigs were adapted to the experimental diets for 5 d and then total but separated collection of feces and urine was conducted for 4 d according to the marker-to-marker procedure (Kong & Adeola, 2014). The collected feces and urine were immediately stored in a freezer at −20 °C prior to further analyses.

### Chemical analysis

At the completion of the study, the frozen fecal samples were dried in a forced-air oven at 55 °C and finely ground prior to chemical analyses. The experimental diets, fecal and urine samples were determined for crude protein (CP) content (N × 6.25) by the Kjeldahl method (Kjeltec 1035; Foss, Hillerod, Denmark).

### Calculations and statistical analysis

Apparent total tract N digestibility and retention were calculated using the following equations:

$${\rm{Apparent}}\;{\rm{total}}\;{\rm{tract}}\;{\rm{N}}\;{\rm{digestibility}}\;\left( {\rm{\% }} \right) = \left( {{{\rm{N}}_{\rm{I}}}{\rm{ - }}{{\rm{N}}_{\rm{F}}}} \right){\rm{/ }}{{\rm{N}}_{\rm{I}}} \times {\rm{100}},$$

$${\rm{N}}\;{\rm{retention}}\;\left( {\% \,{\rm{of}}\,{\rm{intake}}} \right) = \left( {{{\rm{N}}_{\rm{I}}}{\rm{ - }}{{\rm{N}}_{\rm{F}}}{\rm{ - }}{{\rm{N}}_{\rm{U}}}} \right){\rm{/}}{{\rm{N}}_{\rm{I}}} \times {\rm{100}}$$

where: N_I_ is the amount of N ingested (g); N_F_ and N_U_ are the amount of N voided via the feces (g) and urine (g), respectively.

Experimental data were analyzed using the MIXED procedures of SAS (SAS Institute Inc., Cary, NC, USA). The independent variables in the model included diet as a fixed effect and period and block nested within period as random effects. The orthogonal polynomial contrast was used to examine the relationship between N balance response criteria and graded concentrations of Met isomers. The relative bioavailability of D-Met to L-Met was estimated using a multiple regression model and the slope-ratio analysis described by Littell et al. (1997). The statistical model used in the analysis as follows:

$${\rm{y}} = a + \;{b_s}{x_s} + {b_t}{x_t} + e,$$

in which y is response criterion; *a* is intercept; *e* is random error; *b_s_* and *b_t_* are the slopes for L- and D-Met, respectively; *x_s_* and *x_t_* are the concentrations of L- and D-Met intake, respectively. An individual pig served as the experimental unit and statistical significance was determined at *P* < 0.05.

## Results

**Figure 1:**
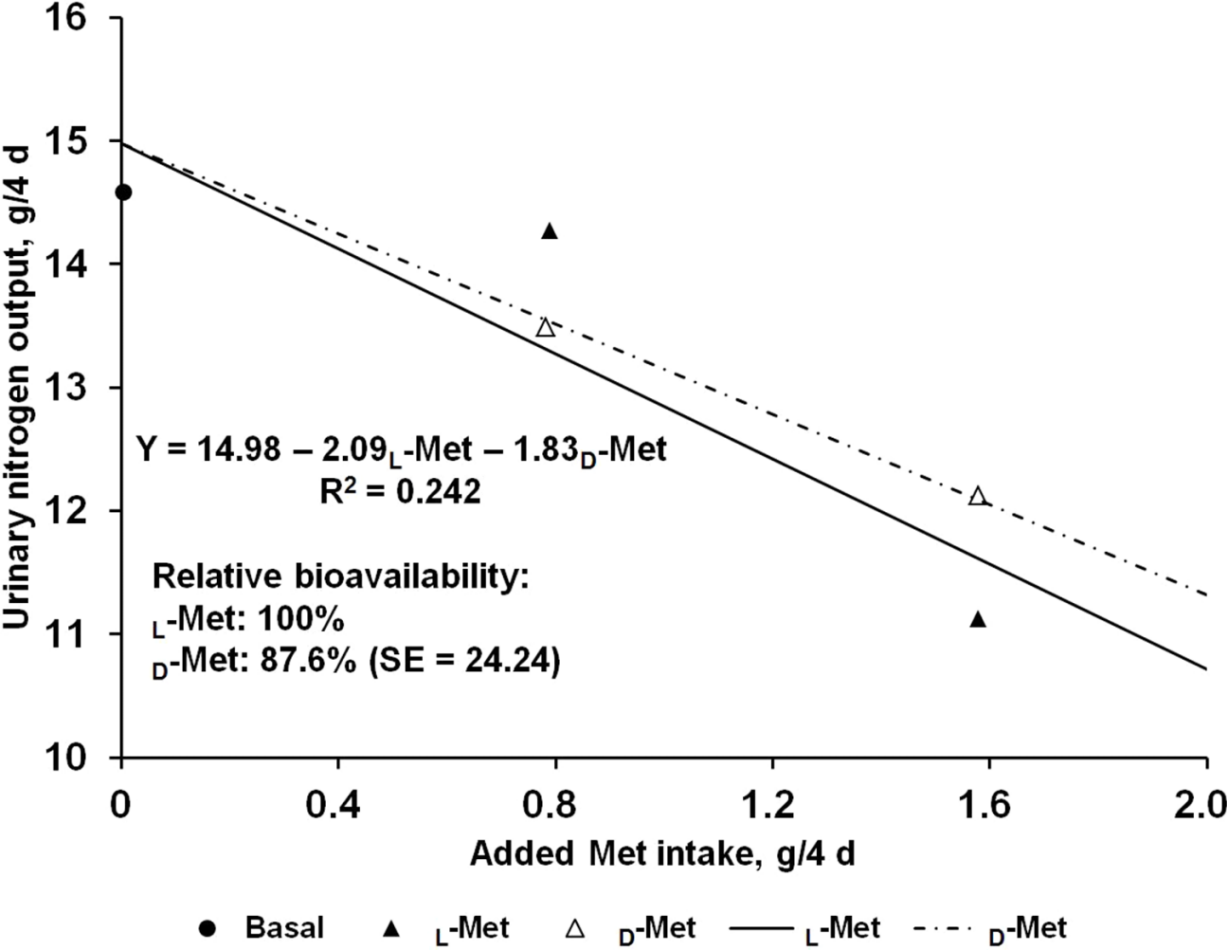
Slope-ratio comparison based on the urinary nitrogen output (g/4 d) of nursery pigs fed diets with graded levels of D-methionine (D-Met) or L-Met. Each data point represents least squares mean of 10 observations except the basal diet (nine observations).

**Figure 2:**
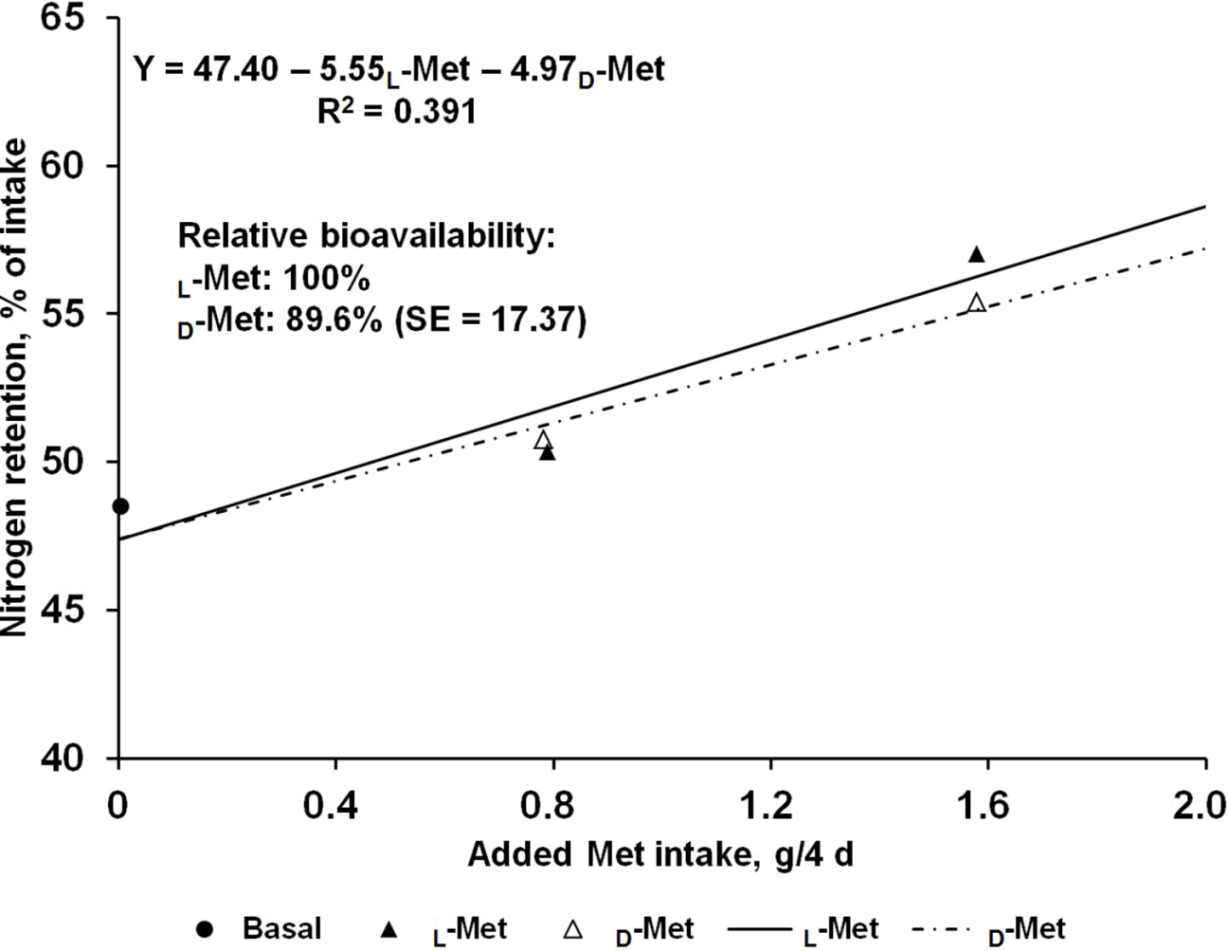
Slope-ratio comparison based on the nitrogen retention (%) of nursery pigs fed diets with graded levels of D-methionine (D-Met) or L-Met. Each data point represents least squares mean of 10 observations except the basal diet (nine observations).

The effects of supplemental L- or D-Met on N balance are shown in Table 2. Nitrogen intakes were similar across the treatments due to the restricted feeding based on the initial BW of pigs. Fecal N output was not affected by Met supplementation regardless of source and consequently apparent N digestibility did not change. In contrast, there was a linear response (*P* < 0.05) to Met supplementation from L- or D-Met in urinary N output, which resulted in increased (*P* < 0.01) retained N and N retention. No quadratic response was observed in any of the N-balance criteria. The estimated bioavailability of D-Met relative to L-Met from urinary N output (g/4 d) and N retention (% of intake) as dependent variables using supplemental Met intake (g/4 d) as an independent variable were 87.6 and 89.6%, respectively (Figs. 1 and 2), but approximate 95% fiducial limits for the relative bioavailability estimates for both dependent variables included 100%.

**Table 2:** Effects of dietary L-methionine (L-Met) and D-Met on nitrogen (N) balance of weaning pigs^†^.

Item	Basal diet	Supplemental L-Met, %		Supplemental D-Met, %		SEM	*P*-values for contrast			
							Linear		Quadratic	
		0.04	0.08	0.04	0.08		L-Met	D-Met	L-Met	D-Met
BW, kg										
Initial	13.9	14.0	14.4	14.0	14.3	0.3	0.116	0.243	0.443	0.733
Final	15.1	15.1	15.6	15.4	15.3	0.4	0.142	0.537	0.277	0.548
Collection period (4 d)										
Feed intake, g	1,973	1,973	1,973	1,955	1,973	40	0.974	0.974	0.985	0.081
N intake, g	44.5	44.5	44.6	44.1	44.6	0.9	0.607	0.607	0.983	0.080
Fecal N output, g	8.33	7.85	8.07	8.24	7.70	0.43	0.630	0.243	0.450	0.612
N digestibility, %	81.3	82.4	82.0	81.4	82.7	0.8	0.537	0.234	0.480	0.555
Urinary N output, g	14.6	14.3	11.1	13.5	12.1	0.6	< 0.001	0.014	0.086	0.862
Retained N, g	21.4	22.4	25.4	22.4	24.8	0.7	< 0.001	< 0.001	0.170	0.337
N retention, % of intake	48.5	50.4	57.0	50.8	55.4	1.2	< 0.001	< 0.001	0.132	0.465

**Note:**

† Each least squares mean represents 10 observations except the basal diet (nine observations).

## Discussion

Nutrient bioavailability assay provides relative information on the capacity of feed ingredients to supply a nutrient capable of being digested, absorbed and available for use or storage (Gabert, Jorgensen & Nyachoti, 2001; Adeola, 2009). Growth performance has generally been used as response criteria for AA bioavailability assay (Chung & Baker, 1992; Adeola, 2009; Shen, Weaver & Kim, 2014). However, in the present study, growth responses to the supplemental Met were not significant among dietary treatments while N balance responses were affected by the supplemental Met. This may be attributed to the relatively greater sensitivity of N balance responses to AA adequacy compared to growth responses in a short-term experiment (Figueroa et al., 2001).

The results from the present study suggest that the relative bioavailability of D- to L-Met, using urinary N output (g/4 d) and N retention (% intake) as dependent variables, are 87.6% and 89.6%, respectively, and the 95% fiducial limits included 100%. To obtain accurate values from bioavailability assays, the assumptions for slope-ratio assay should be validated (Littell et al., 1997). This tested for linearity of the slopes and lack of curvature, and for intersection of responses to reference and test diets at the response to BD. For urinary N output (g/4 d) and N retention (% intake) in the present study, validity tests were performed and all assumptions were valid.

Due to the lack of D-transaminase, pigs are not able to directly utilize D-Met for protein synthesis and S-adenosylmethionine formation. To become bioavailable, D-Met has to be converted to α-keto-γ-methiolbutyrate in a process catalyzed by D-Met oxidase, with subsequent transamination to L-Met (Lewis, 2003). However, the efficiency of these additional enzymatic processes has not been so clear and little information is available on the relative bioefficacy of D- to L-Met. Using phenylalanine as an indicator AA, the relative bioefficacy of D- to L-Met was only 50% when 10 to 14-day-old pigs were used in an indicator AA oxidation study (Kim & Bayley, 1983). Recently, Shen, Weaver & Kim (2014) conducted a relative bioavailability study for a period of 20 d using growth performance as response criteria and reported that the bioavailability of DL- to L-Met was calculated as 69.4 and 81.3% for the average daily gain and gain:feed of nursery pigs, respectively. However, several other studies showed no differences in bioefficacy between D- and L-Met. In the present study, the bioavailability of D-Met was comparable with L-Met in nursery pigs when urinary N output (g/4 d) was used as the response criterion. This was in agreement with Cho et al. (1980) who determined the urinary Met excretion in six-week-old miniature pigs infused with solutions containing L- or DL-Met and reported no difference in utilization between Met isomers. Furthermore, no differences in BW gain or plasma urea levels were observed when three-week-old pigs received low-protein-liquid diets containing either DL- or L-Met (0.51%) for seven days (Reifsnyder, Young & Jones, 1984) and Chung & Baker (1992) reported that D-Met was biologically equivalent to L-Met when the growth performance of pigs averaging 9.6 kg was used as response. It is difficult to explain the reason for discrepancy in the bioefficacy of Met isomers among studies but it may be due in part to the growth stage of animals (Chung & Baker, 1992). The activity of D-Met oxidase, the key enzyme that converts D- to L-Met, was determined to be greater in older animals than in younger animals (D’Aniello et al., 1993). In addition, the methods for measuring bioavailability also affect inconsistent results. As N retention is one of the most sensitive indices for AA utilization (Kim et al., 2006), the N balance technique was employed to determine the relative bioefficacy of D- to L-Met in the present work. In the study conducted by Shen, Weaver & Kim (2014), the bioavailability of DL-Met was less than that of L-Met based on average daily gain and gain:feed, whereas the relative bioavailability of 100.9% was observed for plasma urea N, indicating that the contrary results may be attributed to the use of different response criteria for the estimates of relative bioavailability.

In conclusion, the relative bioavailability values for D- to L-Met in nursery pigs averaging 13.5 kg with the slope-ratio comparison of urinary N output (g/4 d) and N retention (% of intake) were 87.6% and 89.6%, respectively; but 95% fiducial limits for the relative bioavailability estimates included 100%.

## Supplemental Information

10.7717/peerj.2368/supp-1 Supplemental Information 1 Raw Data.

## Additional Information and Declarations

### Data Deposition

The following information was supplied regarding data availability: The raw data has been supplied as Supplemental Dataset Files.

## References

[ref-1] Adeola O (2009). Bioavailability of threonine and tryptophan in peanut meal for starter pigs using slope-ratio assay. Animal.

[ref-2] Cho ES, Andersen DW, Filer LJ, Stegink LD (1980). D-methionine utilization in young miniature pigs, adult rabbits, and adult dogs. Journal of Parenteral and Enteral Nutrition.

[ref-3] Chung TK, Baker DH (1992). Utilization of methionine isomers and analogs by the pig. Canadian Journal of Animal Science.

[ref-4] Cromwell GL (2004). Identifying the limiting amino acids in complex and cereal grain-based diets to minimize nitrogen excretion.

[ref-5] D’Aniello A, D’Onofrio G, Pischetola M, D’Aniello G, Vetere A, Petrucelli L, Fisher GH (1993). Biological role of D-amino acid oxidase and D-aspartate oxidase. Effects of D-amino acids. Journal of Biological Chemistry.

[ref-6] Dibner JJ, Knight CD (1984). Conversion of 2-hydroxy-4-(methylthio)butanoic acid to L-methionine in the chick: a stereospecific pathway. Journal of Nutrition.

[ref-7] Figueroa JL, Lewis AJ, Miller PS, Fischer RS, Gómez RS, Diedrichsen RM (2001). Nitrogen metabolism and growth performance of gilts fed standard corn-soybean meal diets or low-crude protein, amino acid-supplemented diets. Journal of Animal Science.

[ref-8] Gabert VM, Jorgensen H, Nyachoti CM, Lewis AJ, Southern LL (2001). Bioavailability of amino acids in feedstuffs for swine. Swine Nutrition.

[ref-9] Haymond MW (1999). Nutritional and metabolic endpoints. Journal of Nutrition.

[ref-10] Hoehler D, Rademacher M, Mosenthin R (2005). Methionine requirement and commercial methionine sources in growing pigs. Advances in Pork Production.

[ref-11] Kim BG, Lindemann MD, Rademacher M, Brennan JJ, Cromwell GL (2006). Efficacy of DL-methionine hydroxy analog free acid and DL-methionine as methionine sources for pigs. Journal of Animal Science.

[ref-12] Kim K-I, Bayley HS (1983). Amino acid oxidation by young pigs receiving diets with varying levels of sulphur amino acids. British Journal of Nutrition.

[ref-13] Kong C, Adeola O (2014). Evaluation of amino acid and energy utilization in feedstuff for swine and poultry diets. Asian-Australasian Journal of Animal Sciences.

[ref-14] Kong C, Park CS, Ahn JY, Kim BG (2016). Relative bioavailability of DL-methionine compared with L-methionine fed to nursery pigs. Animal Feed Science and Technology.

[ref-15] Lewis AJ (2003). Methionine-Cystine Relationships in Pig Nutrition.

[ref-16] Littell RC, Henry PR, Lewis AJ, Ammerman CB (1997). Estimation of relative bioavailability of nutrients using SAS procedures. Journal of Animal Science.

[ref-17] NRC (1998). Nutrient Requirements of Swine.

[ref-18] Reifsnyder DH, Young CT, Jones EE (1984). The use of low protein liquid diets to determine the methionine requirement and the efficacy of methionine hydroxy analogue for the three-week-old pig. Journal of Nutrition.

[ref-19] Shen YB, Weaver AC, Kim SW (2014). Effect of feed grade L-methionine on growth performance and gut health in nursery pigs compared with conventional DL-methionine. Journal of Animal Science.

